# Ultrasound-mediated blood-brain barrier opening enhances delivery of therapeutically relevant formats of a tau-specific antibody

**DOI:** 10.1038/s41598-019-45577-2

**Published:** 2019-06-25

**Authors:** Phillip W. Janowicz, Gerhard Leinenga, Jürgen Götz, Rebecca M. Nisbet

**Affiliations:** Clem Jones Centre for Ageing Dementia Research, Queensland Brain Institute, The University of Queensland, St Lucia Campus, Brisbane, QLD 4072 Australia

**Keywords:** Blood-brain barrier, Alzheimer's disease

## Abstract

The microtubule-associated protein tau is an attractive therapeutic target for the treatment of Alzheimer’s disease and related tauopathies as its aggregation strongly correlates with disease progression and is considered a key mediator of neuronal toxicity. Delivery of most therapeutics to the brain is, however, inefficient, due to their limited ability to cross the blood-brain barrier (BBB). Therapeutic ultrasound is an emerging non-invasive technology which transiently opens the BBB in a focused manner to allow peripherally delivered molecules to effectively enter the brain. In order to open a large area of the BBB, we developed a scanning ultrasound (SUS) approach by which ultrasound is applied in a sequential pattern across the whole brain. We have previously shown that delivery of an anti-tau antibody in a single-chain variable fragment (scFv) format to the brain is increased with SUS allowing for an enhanced therapeutic effect. Here we compared the delivery of an anti-tau antibody, RN2N, in an scFv, fragment antigen-binding (Fab) and full-sized immunoglobulin G (IgG) format, with and without sonication, into the brain of pR5 tau transgenic mice, a model of tauopathy. Our results revealed that the full-sized IgG reaches a higher concentration in the brain compared with the smaller formats by bypassing renal excretion. No differences in either the ultrasound-mediated uptake or distribution in the brain from the sonication site was observed across the different antibody formats, suggesting that ultrasound can be used to successfully increase the delivery of therapeutic molecules of various sizes into the brain for the treatment of neurological diseases.

## Introduction

Alzheimer’s disease (AD) and related dementias are progressive neurodegenerative diseases for which there is no cure. AD is characterized by the extracellular deposition of amyloid-β (Aβ) as amyloid plaques and the intracellular deposition of tau as neurofibrillary tangles, with the latter directly correlating with dementia in AD patients^1,2^. Reducing tau levels abrogates Aβ-mediated toxicity^3^, making tau an attractive therapeutic target^4^. However, the blood-brain barrier (BBB) limits the passage of molecules from the blood into the central nervous system, and is therefore a formidable obstacle for neurological therapeutics greater than 400 Da, such as immunoglobulin G (IgG) antibodies which are 156 kDa in size. Studies with a therapeutic anti-Aβ antibody have estimated that only around 0.1% of peripherally delivered antibody enters the brain^5,6^. This challenges the therapeutic potential of antibody-based treatments of neurodegenerative diseases and may, at least in part, account for the low clinical success rate of several anti-Aβ therapies^7^. More efficient methods of antibody delivery are therefore essential to increase uptake and reduce potential costs of an antibody-based treatment.

Therapeutic ultrasound is a technique in which biologically inert microbubbles, with a shell of lipid or polymer molecules and a stabilized gas core, are systemically administered and subsequently exposed to non-invasive ultrasound pulses^8,9^. The mechanical interaction between the ultrasound, microbubbles and vasculature transiently opens the tight junctions of the BBB in the sonicated volume, allowing therapeutic molecules to effectively cross this barrier^10,11^. We have previously developed a scanning ultrasound (SUS) approach by which ultrasound is applied in a sequential pattern across the whole brain, which when repeated over several treatment sessions, can reduce plaque load in both middle-aged and very old APP23 transgenic mice^12,13^. More recently, we combined the administration of an anti-tau antibody, RN2N, in a single-chain variable fragment (scFv) format with SUS and demonstrated a significant increase in the delivery of the scFv to the brain of P301L tau transgenic pR5 mice, a model of tauopathy^14^. Furthermore, treatment of the pR5 mice with the RN2N scFv delivered by SUS produced a significant reduction in pathological tau phosphorylation and anxiety-like behaviour compared to scFv only-treated mice^14^. This study demonstrated that SUS can enhance the delivery of an anti-tau scFv into the brain, thereby providing an enhanced therapeutic effect.

Our work and that of others demonstrate a growing interest in the use of therapeutic ultrasound for the delivery of full-length and fragment antibodies to overcome the BBB for therapeutic purposes^15,16^. It is therefore important to characterize ultrasound-mediated delivery of these antibodies and determine the impact of antibody format. Delivery studies using dextrans of varying sizes have shown that with a 0.56 MPa peak acoustic pressure, a higher concentration of 3 kDa dextran is delivered to the brain compared with 70 kDa dextran^17^. Biologically, however, antibodies differ significantly from dextrans in both their shape and flexibility, as well as their high target specificity and effector functions, all of which may affect brain uptake in addition to size. This study therefore aimed to compare the delivery of the tau-specific antibody, RN2N, in an scFv, fragment antigen-binding (Fab) or full-sized murine IgG format, to determine the importance of antibody format for enhanced ultrasound-mediated brain delivery. We show that although the IgG reaches a higher concentration in the brain in comparison with the smaller formats by bypassing renal excretion, no differences in either the ultrasound-mediated uptake or diffusion from the sonication site were observed between the formats.

## Results

### SUS enhances antibody delivery to the brain

**Figure 1:**
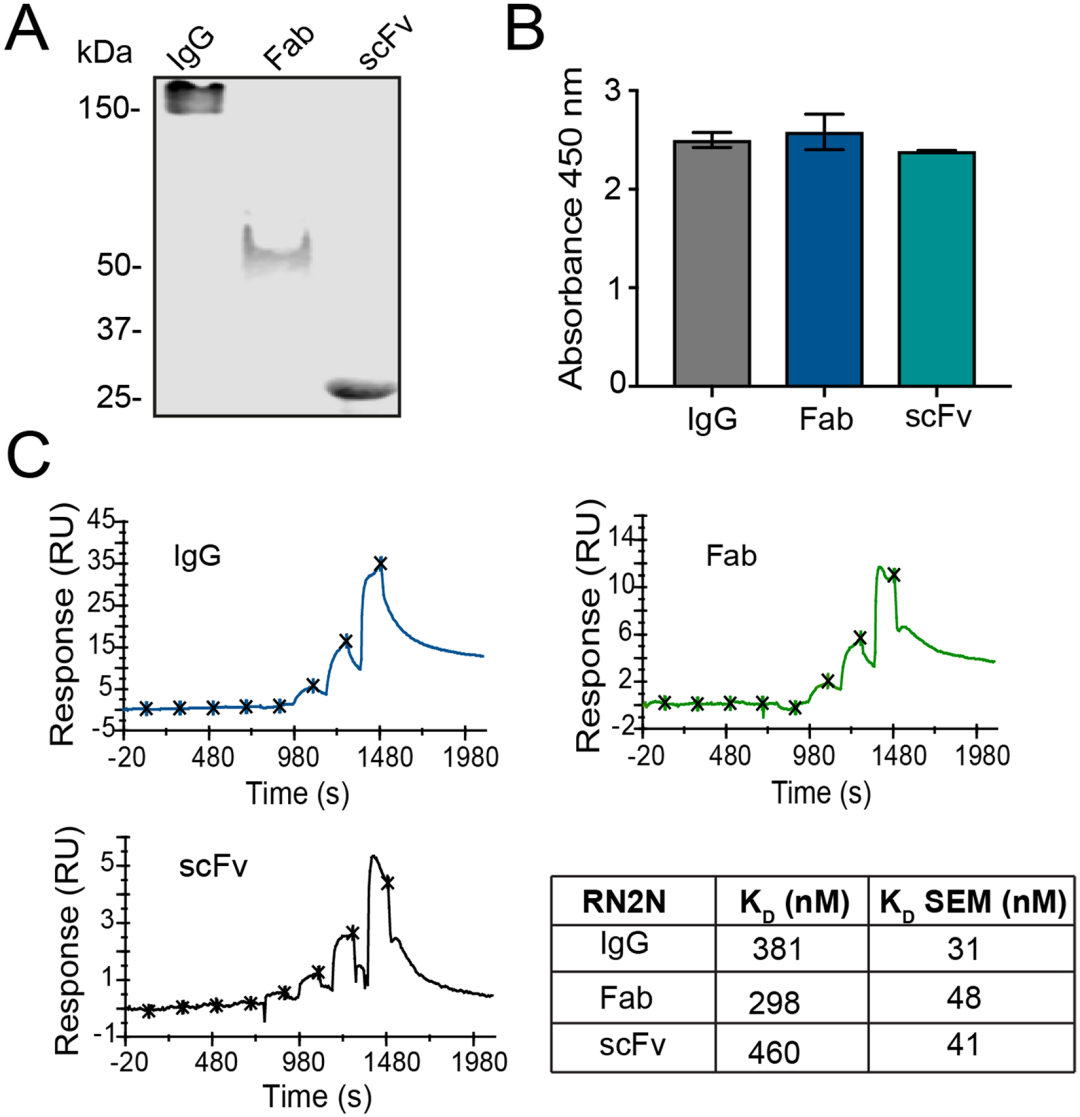
RN2N specificity and binding are retained in all antibody formats. (**A**) Gel electrophoresis and Coomassie staining of purified antibody formats shows that the 2N tau-specific RN2N antibody in an IgG format is approximately 156 kDa in size, the Fab is approximately 52 kDa in size and the scFv is approximately 29 kDa in size. (**B**) RN2N in all formats was shown to bind to full-length human tau using ELISA. (**C**) The single-cycle kinetics of RN2N to full-length human tau was determined using surface plasmon resonance. The *K*_D_ of RN2N IgG was 381 nM, consistent with Fab (298 nM) and scFv (460 nM).

We have previously demonstrated that the RN2N anti-tau antibody in the scFv format, when combined with SUS, reaches a higher concentration in the brain producing an enhanced therapeutic effect in P301L tau transgenic pR5 mice^14^. This mouse strain expresses the longest isoform of human tau, 2N4R, and is characterized by progressive tau pathology in the brain^18^. Here, our goal was to determine whether antibody properties, including size, affinity and Fc receptor binding, are important for effective brain delivery. To achieve this, full-sized murine IgG2a (156 kDa), Fab (52 kDa) and the previously characterized scFv (29 kDa) were generated (Fig. 1A; Supplementary Fig. 1). To ensure that binding to tau was retained in all formats, an ELISA was performed against human tau, which revealed that engineering of the antibody did not negatively impact its binding (Fig. 1B). Furthermore, the binding affinity to tau, as determined by single-cycle surface plasmon resonance, did not differ significantly between the antibody formats, falling within a range of 298–460 nM (Fig. 1C).

**Figure 2:**
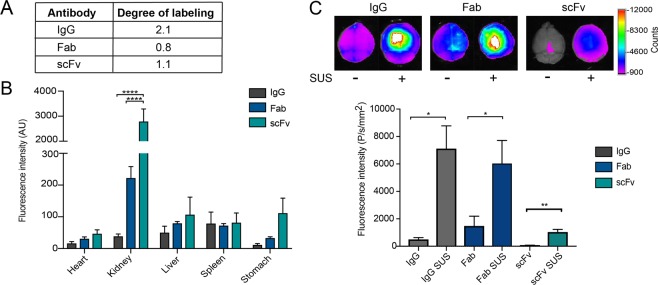
SUS enhances delivery of all RN2N antibody formats across the BBB. (**A**) Degree of Alexa-647 labeling of the antibody formats. (**B**) Quantification of Alexa Fluor 647-conjugated RN2N fluorescence intensity in peripheral organs of sonicated 2N tau-overexpressing pR5 mice using SUS (scanning ultrasound). The scFv produced a greater signal in the kidneys when compared to the IgG and Fab, whereas the other organs did not differ significantly (n = 5; mean ± SEM, two-way ANOVA with Tukey’s multiple comparisons test, ****P < 0.0001). (**C**) Alexa Fluor 647-conjugated RN2N was delivered to pR5 mice using SUS and including sham (microbubble injection, but no ultrasound treatment) as control. Brains were imaged with a Bruker *In Vivo* MS FX Pro optical imaging system with a 630 nm excitation filter and a 700 nm emission filter, and the fluorescence intensity was quantified and normalized to the degree of labeling. The fluorescence intensity of the IgG and Fab was greater than that of the scFv for both with or without SUS. The fluorescence intensity of all antibody formats was enhanced when delivery was combined with SUS (n = 5, mean ± SEM; student t-test, *P < 0.05, **P < 0.01).

We next sought to compare the delivery of the different antibody formats to the brain with and without SUS in the pR5 mice. To detect the antibodies post-treatment in the pR5 mice, antibodies were labeled with Alexa-647 and the degree of labeling was calculated (Fig. 2A). The first cohort of mice was split into six treatment groups, receiving equal moles of Alexa-647-labeled IgG, Fab or scFv by retro-orbital injection, and then being subject to SUS or sham treatment (microbubble injection without applying ultrasound). Following treatment, the mice were perfused to remove the antibody from their vasculature, and their organs were harvested and analyzed. A significant increase in the level of the scFv was observed in the kidney compared to Fab and IgG, whereas the signal in the other peripheral organs (heart, liver, spleen and stomach) did not differ significantly (Fig. 2B). Analysis of the whole brain fluorescence intensity revealed minimal antibody fluorescence in the sham-treated brains (Fig. 2C). In the SUS-treated mice, however, brain antibody fluorescence was significantly increased for all formats (Fig. 2C), demonstrating that SUS can enhance the brain delivery of antibody formats ranging in size from 29 to 156 kDa.

### Full-sized IgG antibodies reach a higher concentration in the brain following SUS

**Figure 3:**
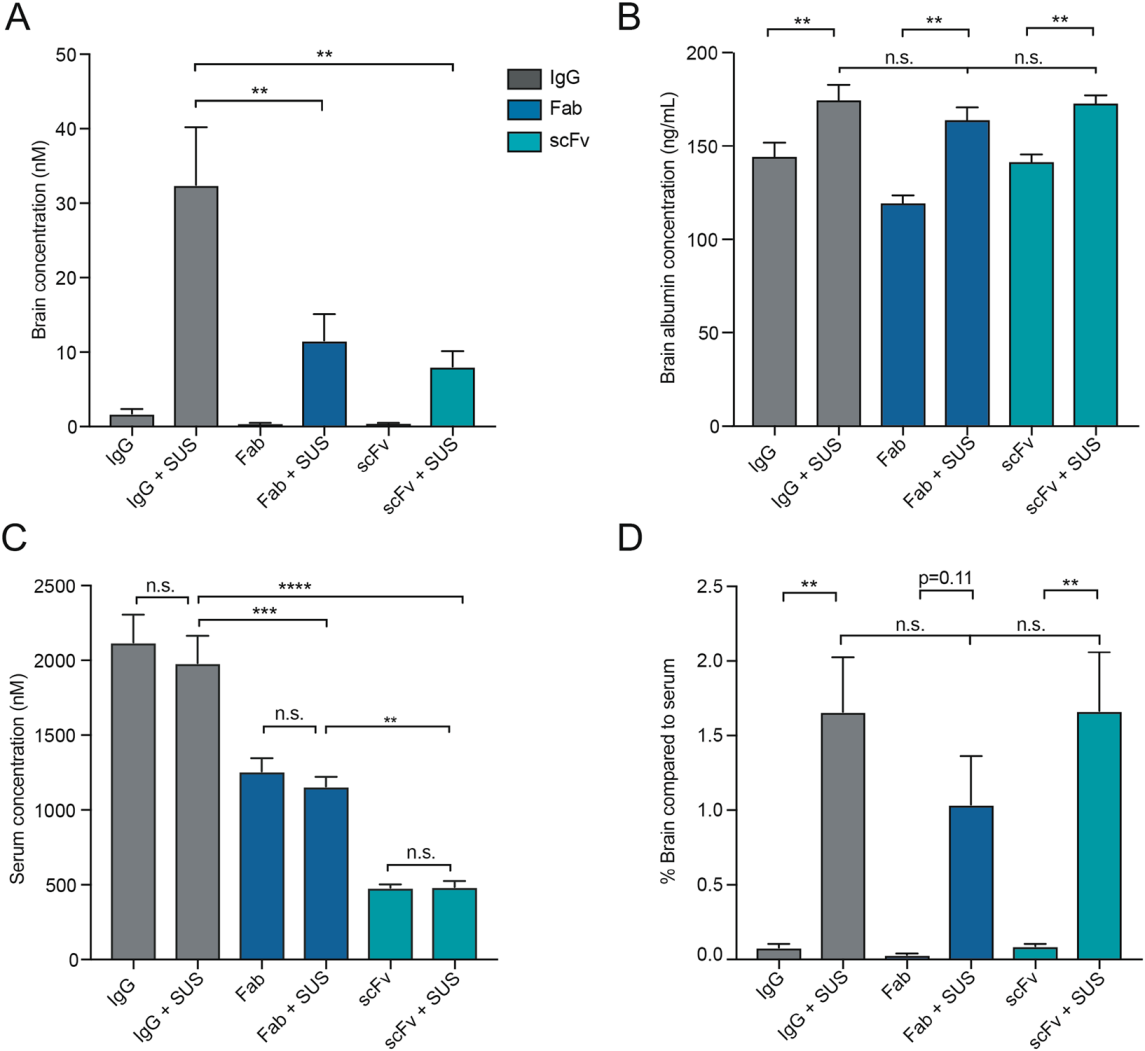
Full-sized IgG antibodies reach a higher concentration in the brain following SUS treatment. (**A**) Determination of RN2N concentration in the brain post-delivery. SUS treatment increased the mean concentration of all formats in the brain (19-fold for IgG, 30-fold for Fab and 20-fold for scFv). Furthermore, following SUS, the concentration of the IgG was significantly increased compared to that of scFv and Fab. (**B**) SUS treatment increased the concentration of albumin in the brain, but there were no significant differences in albumin concentration in the brains treated with the different RN2N formats. (**C**) No significant difference in the serum concentration of RN2N was observed between mice treated with or without SUS. Serum concentrations of the IgGs and Fab formats were significantly higher than that of the scFv, in both the SUS- and sham-treated groups. (**D**) The percentage concentration of the antibody formats in the brain after SUS delivery compared to that in the serum was consistent between the different formats demonstrating that brain delivery following SUS was proportional to the corresponding serum levels. (n = 5, mean ± SEM; one-way ANOVA with Tukey’s multiple comparisons test; **P < 0.01, ***P < 0.001, ****P < 0.0001).

As the antibody formats have differing degrees of labeling and therefore cannot be accurately compared by imaging, the concentrations of the antibody formats (in brain homogenates and serum) were determined by comparing the fluorescence intensity of the antibodies in the brain and serum to that of standards for each format (Supplementary Figs 2 and 3). The brain concentration of the antibodies in the sham-treated groups reached the lower detection limit of the assay and were variable (Fig. 3A). However, the concentrations of the antibody formats in the SUS-treated groups were higher, and there was significantly more IgG in the brain (32 nM) compared to the Fab and scFv formats (12 nM and 8.1 nM, respectively) (Fig. 3A). To ensure that the difference in concentration of the different formats in the brain was not due to variations in the amount of BBB opening between each group, the concentration of albumin was measured in the brain of the treated mice (Fig. 3B). Comparison of the sham-treated mice to the SUS-treated mice for all antibody formats revealed a significant increase in the concentration of albumin in the SUS-treated mice, consistent with BBB opening in this group (Fig. 3B). This is consistent with our previous work showing that Evans blue-bound albumin (sized 66 kDa) can enter the brain following SUS treatment^12^. However, there was no significant difference in the concentration of albumin between the SUS-treated groups, demonstrating that there was a comparable degree of BBB opening for each antibody format. Taken together, these findings suggest that the differences in antibody concentration (Fig. 3A) are only due to the serum levels of the antibody and not to the degree of BBB opening. As the scFv and Fab formats demonstrated an increased localization to the kidneys (Fig. 2B), we also investigated whether the reduced concentration of scFv and Fab in the brain was due to a decrease in their circulating levels in the blood. Analysing the serum concentration of each of the antibodies revealed that the concentrations of scFv and Fab, either with or without SUS, were significantly lower than that of IgG (Fig. 3C), which may be attributable to the increased clearance of these formats through the renal system. Despite reduced circulating scFv and Fab, the percentage concentration of the antibody formats in the brain after SUS delivery compared to that in the serum was consistent between the different formats (Fig. 3D) demonstrating that their delivery into the brain following SUS was proportional to their serum level and suggesting that SUS efficiency is the same for all antibody sizes.

### Determination of antibody distribution in the brain after delivery

**Figure 4:**
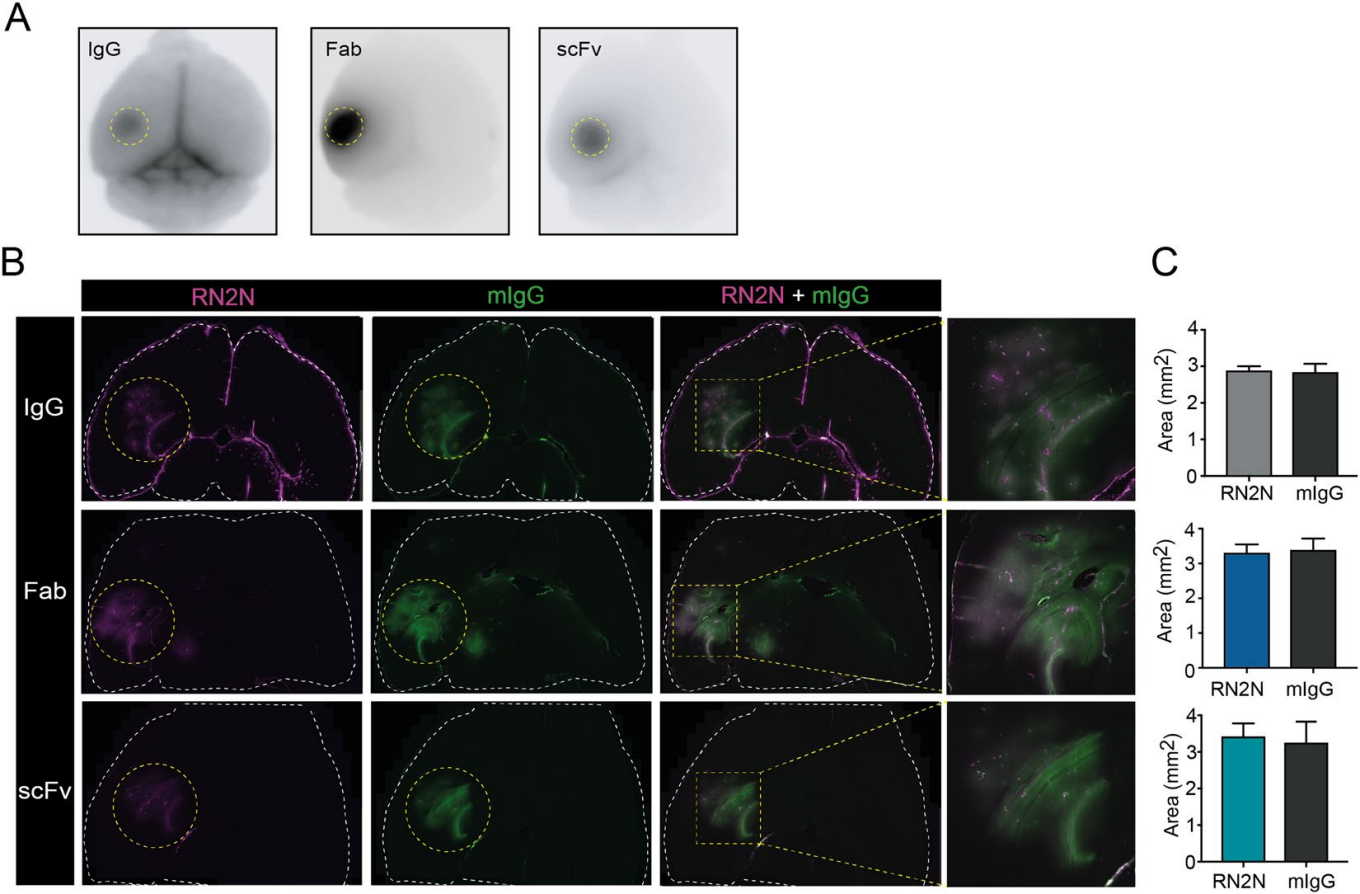
RN2N antibody formats show similar brain distribution following ultrasound treatment of a volume comprising the hippocampus. (**A**) Alexa Fluor 647-conjugated RN2N fluorescent signal after a sonication targeting the hippocampus. (**B**) Co-localization of the RN2N fluorescence with endogenous mouse IgG (mIgG) in the targeted hippocampus. (**C**) Calculation of the area distribution of RN2N and endogenous mouse IgG revealed no significant difference between the different sized RN2N antibodies compared to mouse IgG. (n = 3, mean ± SEM; student t-test).

It has previously been demonstrated that smaller dextrans (3 kDa) distribute more diffusely following sonication than larger dextrans (70 kDa)^17^. To investigate antibody distribution following BBB opening and to determine whether this was affected by the antibody format, ultrasound was only targeted to the hippocampus (Fig. 4A) as previously described (i.e. not using a SUS mode)^17^. Equal moles of Alexa-647 dye (conjugated to the antibodies) were injected to account for different degrees of antibody labeling. Using previously reported coordinates^17^, we were able to target the entire hippocampus and parts of the adjacent cortex and lateral ventricle (Fig. 4B). To determine if antibody size had an effect on distribution at the site of sonication, the signal of the RN2N formats was compared with that of endogenous mouse IgG used as an internal control for brain uptake (Fig. 4B). This revealed that RN2N in all formats co-localized with the mouse IgG (Fig. 4B). Furthermore, no significant differences were observed in the total area of antibody distribution between the RN2N formats and mouse IgG (Fig. 4C), demonstrating that the antibody format did not have an effect on the distribution of the antibody at the site of sonication.

## Discussion

The BBB presents a major hurdle for the treatment of neurological disorders^19,20^. It is therefore imperative to establish techniques which enhance the delivery of therapeutic molecules across this barrier, and to determine the biophysical parameters which make therapeutics amenable to this mode of transport. Using the anti-tau antibody, RN2N, as a model antibody^14^, we directly compared the SUS-mediated delivery of antibodies with the same specificity and affinity but in different formats to determine the effect of antibody format on delivery into the brain.

We found that all fluorescently conjugated antibody formats (29–156 kDa) crossed the BBB after sonication, as demonstrated by an increase in fluorescence intensity in the sonicated areas. Despite its comparably large size, we observed that RN2N IgG achieved the highest concentration in the brain. This was not due to differences in the amount of uptake following sonication, as there were no significant differences in the ratio of antibody levels in serum compared to brain. Rather, the effect was due to the longer half-life of the full-sized antibody, which led to an increase in the circulating serum levels of the IgGs compared to the smaller Fab and scFv formats^21^.

Although the smaller antibody formats reached a lower concentration in the brain following sonication, the distribution of the various formats throughout the targeted area (i.e. the hippocampus) did not differ from that of endogenous mouse IgG, suggesting that antibody format does not affect distribution throughout the target area. Nonetheless, it is worthwhile considering that this study was conducted using a transgenic mouse model which over-expresses human tau, and that the presence of this tau may have influenced the delivery of RN2N across the BBB and its diffusion throughout the target area. Furthermore, intracellular tau aggregates within the brain have previously been shown to enhance brain uptake of tau-specific antibodies from the periphery and into neurons^22,23^. As we observed similar diffusion patterns of the RN2N and endogenous mouse IgG, our results suggest that ultrasound-mediated delivery of antibodies across the BBB and their diffusion throughout the targeted area are not dependent on the presence of the antigen. Characterizing the antibody signal at later timepoints post-sonication delivery will, however, determine if the presence of antigen is required for the retention of the delivered antibody in the brain as well as cellular uptake.

In conclusion, we have shown that antibody format does not have an effect on the efficiency of ultrasound-mediated delivery across the BBB, or the distribution throughout the target area, demonstrating that this technique is a valuable strategy for enhancing the delivery of antibody formats across a wide size range to the brain. As the full-sized antibody format persisted longer in the serum than the scFv or Fab and therefore reached a higher concentration in the brain; however, this suggests that this format may be therapeutically advantageous for the treatment of neurological diseases.

## Materials and Methods

### Antibody generation

RN2N is a mouse monoclonal antibody raised against the tau peptide TEIPEGITAEEAGI and is specific for the 2N isoforms of tau^14,24,25^. The IgG and scFv were generated as previously described^14^. The IgG isotype used for this study was IgG2a. To produce the Fab, purified IgG was digested with papain using the Pierce™ Fab Preparation Kit (Thermo Fisher Scientific). The calculated molecular weight of the Fab was 52 kDa. The scFv with a C-terminal His6 and myc tag was expressed in BL21 cells and purified as previously described^14^. All antibody formats were stored in 1X PBS (137 mM NaCl, 2.7 mM KCl, 10 mM Na_2_PO_4_, 1.8 mM KH_2_PO_4_) at −80 °C. To assess RN2N scFv and Fab generation, proteins were electrophoresed on a 10% Tris-glycine SDS-PAGE gel and then stained with Coomassie blue R250 (Biorad). The scFv was generated as previously described^14^ and was approximately 29 kDa in size.

### Recombinant tau generation

cDNA encoding full-length human tau (Tau 441) was cloned into pET-DEST42 vector (Thermo Fisher Scientific) in frame with a C-terminal His6 and V5 tag. Plasmids were transformed into One Shot® BL21™ bacterial cells (Thermo Fisher Scientific) and recombinant protein expression was induced with 1 mM IPTG for 2 hrs at 37 °C. The bacterial suspension was pelleted at 4,000 × g for 15 min at 4 °C then resuspended in IMAC buffer [300 mM KCl, 50 mM KH_2_PO_4_, 5 mM Imidazole, pH 8.0] containing 0.1% Complete protease inhibitor and 0.1 mg/ml Lysozyme (Sigma) followed by incubation on ice for 20 min. The cells were then subjected to repeated freeze/thawing then sonicated at 60% amplitude for 1 minute (Qsonica Sonicator). The lysate was centrifuged at 16,000 × g for 20 min at 4 °C and filtered through a 0.22 μm syringe filter (Millipore). The filtered lysates were passed over a 1 ml Bio-Scale Mini IMAC cartridge (Bio-Rad) equilibrated in IMAC buffer and eluted in 300 mM KCL, 50 mM KH_2_PO_4_, 250 mM Imidazole, pH 8.0. Eluted proteins underwent buffer exchange into 137 mM NaCl, 2.7 mM KCl, 4.3 mM Na_2_HPO_4_, 88.1 mM KH_2_PO_4_, pH 7.4 using a Bio-Scale Mini Bio-Gel P-6 Desalting Cartridge (Bio-Rad) and were then subjected to size exclusion chromatography using an S200 10/30 GL column (GE Healthcare) equilibrated in 1 X PBS. Fractions corresponding to the protein of interest were combined and concentrated using an Amicon® Ultra Filter (Merck Millipore).

### ELISA

The binding specificity of the RN2N antibody formats was analysed using an enzyme-linked immunosorbent assay (ELISA) as previously described^25^. Briefly, Immuno 96 MicroWell plates (Nunc) were coated overnight at 4 °C with recombinant tau at 10 μg/mL in PBS. The plates were washed then blocked with 3% BSA in PBS. RN2N antibody formats were incubated at 10 μg/mL in PBS and then washed with PBS. ScFv binding was detected following incubation with an anti-Myc antibody (1:1000; Cell Signalling Technologies), washed as above and then incubated with an anti-mouse horse-radish peroxidase antibody conjugate (1:5000; Thermo Fisher Scientific). Fab and IgG binding were detected following incubation with the anti-mouse horse-radish peroxidase antibody conjugate with a wash in between. Substrate solution, 1-Step Ultra TMB (Thermo Fisher Scientific), was added to each well, after which the reaction was stopped with 1 M HCl. The absorbance was measured with a 450 nm filter (CLARIOstar, BMG Labtech). Measurements were performed in triplicate and analyzed after background subtraction.

### Surface plasmon resonance

Surface plasmon resonance measurements were conducted at the Monash Fragment Platform, Monash University, using the Biacore S200 biosensor (GE Healthcare). Biotinylated RN2N was captured on a streptavidin-coated CM5 chip (GE Healthcare). For biotinylation, RN2N IgG1 (29.5 μM), RN2N IgG2a (32.0 μM), RN2N Fab (22.9 μM) or RN2N scFv (60.4 μM) in PBS was added in a 1:1 ratio with EZ-link NHS-LC-LC-biotin (Thermo Fisher Scientific) and incubated at 25 °C for 1 h. Antibodies were separated from free-unconjugated biotin using size-exclusion chromatography on a Superdex 200 10/300 GL (GE Healthcare) or Superdex 75 10/300 Increase (GE Healthcare) equilibrated in PBS. Streptavidin was immobilized on the CM5 chip using amine coupling at 37 °C. Antibodies were captured at 25 °C, using a flowrate of 10 µL/min, in PBS for IgG and Fab or immobilization buffer 2 (12 mM Na_2_HPO_4_, 287 mM NaCl, 2.7 mM KCl, 1.8 mM KH_2_PO_4_, 0.05% Tween-20 pH 7.4) for the scFv. Tau binding experiments were run using single-cycle kinetics at 25 °C with the running buffer (12 mM Na_2_HPO_4_, 287 mM NaCl, 2.7 mM KCl, 1.8 mM KH_2_PO_4_, 0.05% Tween-20 pH 7.4). Tau was injected for 120 s at a flow rate of 40 µL/min with a dissociation time of 600 s, using 8 concentrations of tau (1/3 serial dilutions from 0.0128–1,000 nM). The data were processed using Biacore S200 Evaluation Software Version 1.0, double referenced against blank injections of buffer and fit to a Steady State Affinity model using report points 4 s before the injection end, with a 5 s window.

### Antibody labeling

RN2N was covalently conjugated with Alexa Fluor 647 dye (Thermo Fisher Scientific) in PBS with 0.1 M sodium bicarbonate, as described previously^14^. The labelled antibodies were then separated from free dye using a Superdex 200 10/300 column (GE Healthcare) equilibrated in 1 X PBS, pH 7.4, at 0.5 mL/min, and the degree of labeling was determined. Antibody-Alexa Fluor 647 conjugates were then concentrated to the desired injection volumes using an Amicon® Ultra filter (Millipore).

### Mice

All animal experiments were conducted under the guidelines of the Australian Code of Practice for the Care and Use of Animals for Scientific Purposes and were approved by the University of Queensland Animal Ethics Committee (QBI/412/14/NHMRC; QBI/554/17/NHMRC). Three to six month old, pR5 mice which express 2N4R tau with the P301L mutation under the control of the mThy.1.2 promoter^18^ were used for the study.

### Production of microbubbles

Microbubbles comprising a phospholipid shell and octafluoropropane gas core were generated in-house as previously described^12^.

### Scanning ultrasound

For SUS experiments, 6-month-old pR5 mice were randomly assigned to one of the following groups: SUS only, antibody only, or SUS and antibody combined. Five mice were used per experimental group. 24 h prior to treatment, animals were anaesthetized with ketamine (100 mg/kg) and xylazine (10 mg/kg), their head shaved and residual hair removed using hair removal cream. Immediately prior to treatment, all animals were anaesthetized again and prepared as previously described^12^. For the SUS-only group, mice were injected retro-orbitally with 40 μL of microbubble solution prepared as previously described^12^. For the antibody only and antibody plus SUS groups, 3 nanomoles of each antibody and 40 μL microbubble solution were mixed in a 29 G 0.5 mL insulin syringe (Terumo), incubated in-hand for 15 s to bring to body temperature and then injected retro-orbitally. The maximal combined injection volume was 120 µL. Animals which received SUS were placed in a head frame (Narishige) and SUS was applied to the entire brain as previously described^12^. Briefly, SUS was conducted using the Therapy Imaging Probe System (TIPS, Philips Research) with the following settings: 1 MHz centre frequency, 0.65 MPa peak rarefactional pressure applied outside the skull, 10 Hz pulse repetition frequency, 10 ms pulse length and a 10% duty cycle. The focus of the transducer had dimensions of 1.5 mm × 12 mm in the transverse and axial planes, respectively. The motorized positioning system moved the focus of the transducer array in a grid with 1.5 mm spacing between individual sites of sonication so that ultrasound was delivered sequentially to the entire brain with a 6 s duration sonication per spot. After 30–60 min, blood was collected from the anaesthetized mice using an EDTA-coated 27 G syringe (Nipro) in the right ventricle, after which they were immediately transcardially perfused with ice-cold PBS using a 23 G butterfly syringe (Terumo) via the left ventricle, and their brains harvested. The dissected brains were then immediately imaged in the Bruker MS FX Pro on a petri dish (Falcon), after which the hemispheres were separated, with one hemisphere being snap-frozen in liquid nitrogen, and the other immersion-fixated in 4% paraformaldehyde (Merck). Blood was centrifuged at 1500 × g for 10 min at 4 °C, and serum collected.

### Ultrasound targeting of the hippocampus

For only targeting the hippocampus, the same equipment and protocols were used as for SUS, except that 3-month-old pR5 mice were anaesthetized using 1–2% isoflurane, and a single hippocampal spot was targeted for 60 s at 0.6 MPa (Fig. 4A). The skin was gently held taut using electrical tape, and the lambdoid and sagittal sutures were lightly marked with pen as a crosshair to from which to move the transducer. Ultrasound was applied at coordinates of 2 mm anterior to the lambdoid suture, and 2.25 mm laterally (to the left hand-side), as described in previous literature^17^. Equal moles (3 nanomoles) of dye per antibody injection (instead of equal moles of antibody) were injected to eliminate any differences from unequal degrees of labeling.

### Imaging

Imaging of the SUS-treated brains was performed using a Carestream MS FX Pro (Bruker) with 630 nm excitation filter, and 700 nm emission filter, at 10 s exposure time and signal intensity was measured using the Molecular Imaging (MI) software (Bruker). Imaging of the hippocampal-treated brains and peripheral organs were acquired with an Odyssey Fc (LI-COR Biosciences) using the 700 nm channel and signal intensity was measured using the Image Studio software (LI-COR Biosciences).

### Determination of brain and blood antibody concentrations

The snap-frozen brain hemispheres were weighed and diluted 3X (m/v) in RIPA buffer (Cell Signalling) with complete protease inhibitor tablet (Sigma-Aldrich), homogenized and lysed by centrifugation at 13000 × g for 90 min at 4 °C. Alexa Fluor 647 fluorescence intensity in the soluble RIPA fraction of brain homogenate was measured using a ClarioStar Fluorescent plate reader (BMG Labtech) and compared to standard curves (Supplementary Fig. 2) of Alexa Fluor 647-conjugated antibody-spiked brain homogenate. The serum concentration was calculated in the same way, except that control mouse serum was used for the standard curves (Supplementary Fig. 3).

### Determination of brain albumin concentration

Lysed brain samples from above were analysed using a mouse albumin ELISA kit (Abcam) following the manufacturer’s instructions.

### Histology

Fixed brain hemispheres were cryo-protected by immersion in 30% sucrose for 48 h at 4 °C and then sectioned at 40 μm thickness horizontally on a freezing sliding microtome with dry ice and FSC22 cryoprotectant (Leica). For immunofluorescence, every 6th section (200 μm apart) from the superior to inferior hippocampus was incubated overnight with anti-mouse IgG Alexa-488 antibody (1:500; Thermo Fisher Scientific), then cover-slipped with fluorescence mounting medium (Dako) and sealed using nail polish. Images were then captured with a fluorescence slide scanner (Zeiss) with Metafer4 software (Metacyte).

### Statistical analysis

Statistical analyses were performed with GraphPad Prism 7.0 software using one-way ANOVA with Tukey’s multiple comparison test for data with multiple groups or an unpaired t-test for data comparing two groups. All values are given as the mean ± standard error of the mean (SEM). Five mice were used per group for the SUS experiment, whereas three to four were used per group for the hippocampus-targeted experiment.

## Supplementary information

Supplementary Dataset 1

## Data Availability

The datasets generated during and/or analysed during the current study are available from the corresponding author on reasonable request.
