# Supplementary material for: Ultrasound-mediated blood-brain barrier opening enhances delivery of therapeutically relevant formats of a tau-specific antibody
Source: Sci Rep. 2019 Jun 25;9:9255. doi: 10.1038/s41598-019-45577-2 (PMC6592925; doi:10.1038/s41598-019-45577-2)
Supplement: Supplementary file 1 — Supplementary Dataset 1 [file 41598_2019_45577_MOESM1_ESM.pdf]

Ultrasound-mediated blood-brain barrier opening enhances delivery of therapeutically relevant formats of a tau-specific antibody

Phillip W. Janowicz[1], Gerhard Leinenga[1], Jürgen Götz[1#], Rebecca M. Nisbet[1#]

[1] Clem Jones Centre for Ageing Dementia Research, Queensland Brain Institute, The University of Queensland, St Lucia Campus, Brisbane, QLD 4072, Australia

# Correspondence to: Rebecca Nisbet

E-mail: r.nisbet@uq.edu.au

Correspondence may also be addressed to: Jürgen Götz

E-mail: j.goetz@uq.edu.au

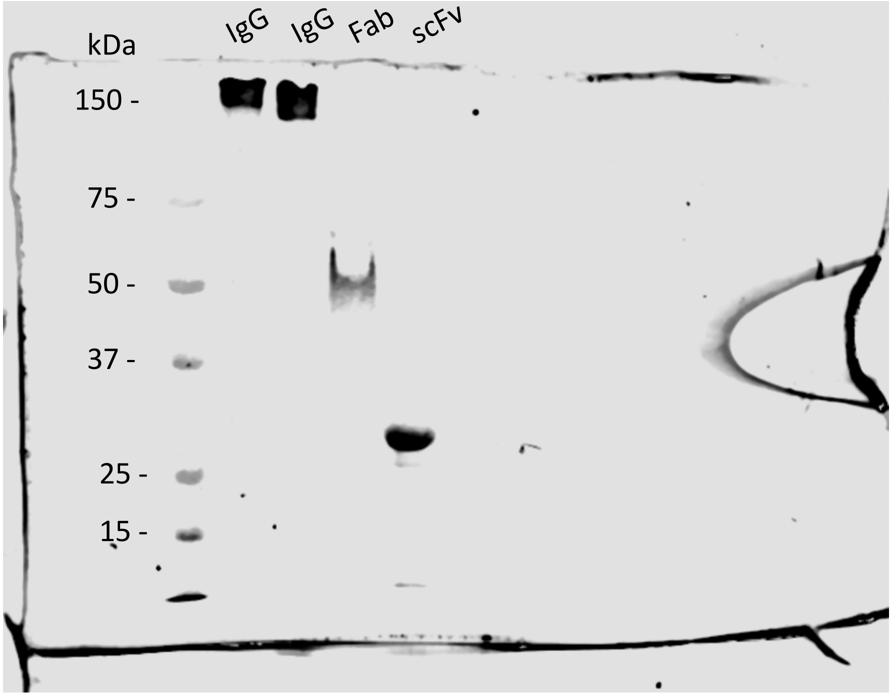

Supplementary Figure 1. Full-sized gel of electrophoresed and Coomassie stained purified RN2N antibody formats. IgG is approximately 156 kDa in size, the Fab is approximately 56 kDa in size and the scFv is approximately 29 kDa in size.

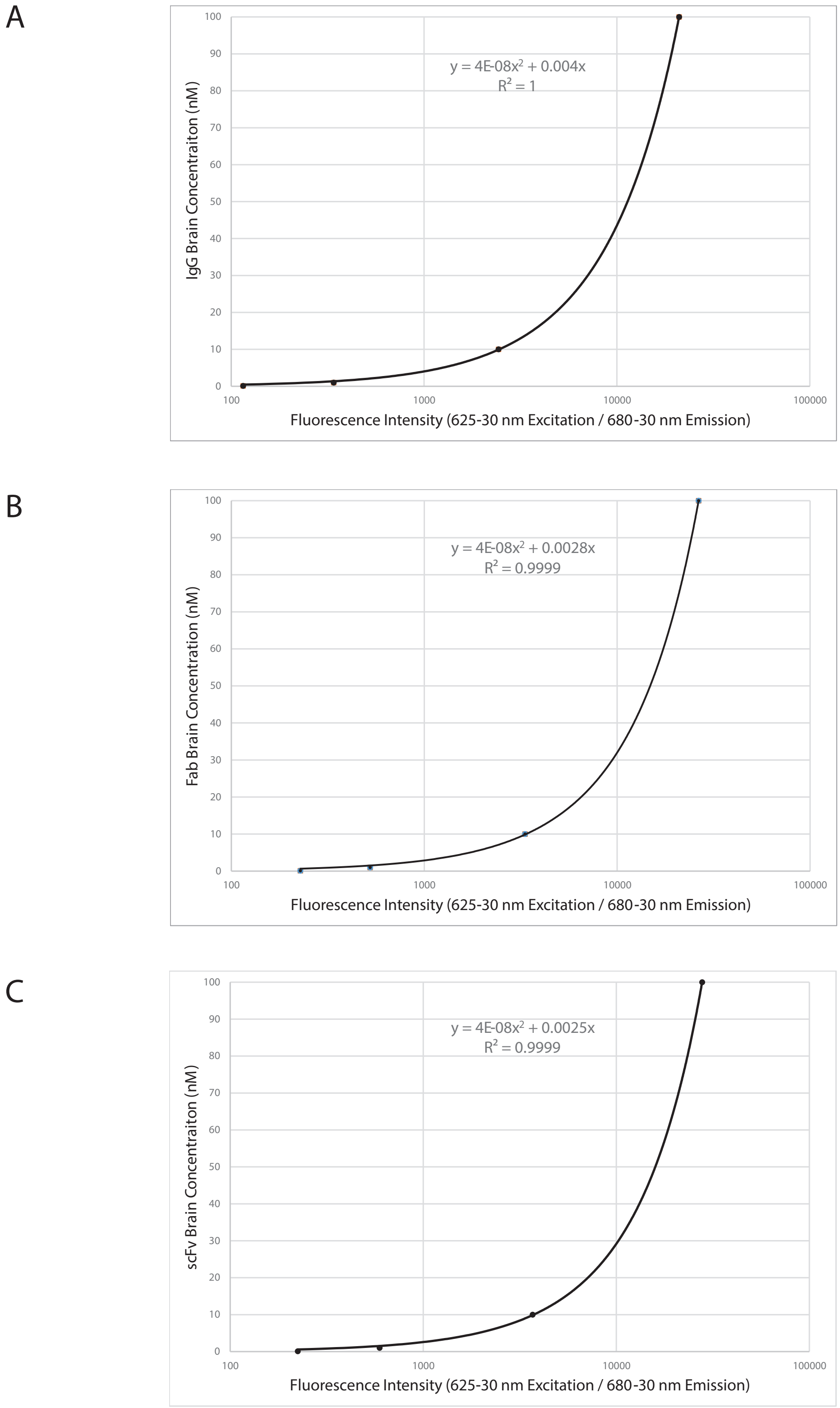

Supplementary Figure 2: Standard curves of fluorescence intensity used to calculate RN2N (A) IgG, (B) Fab (B), and (C) scFv brain concentration.

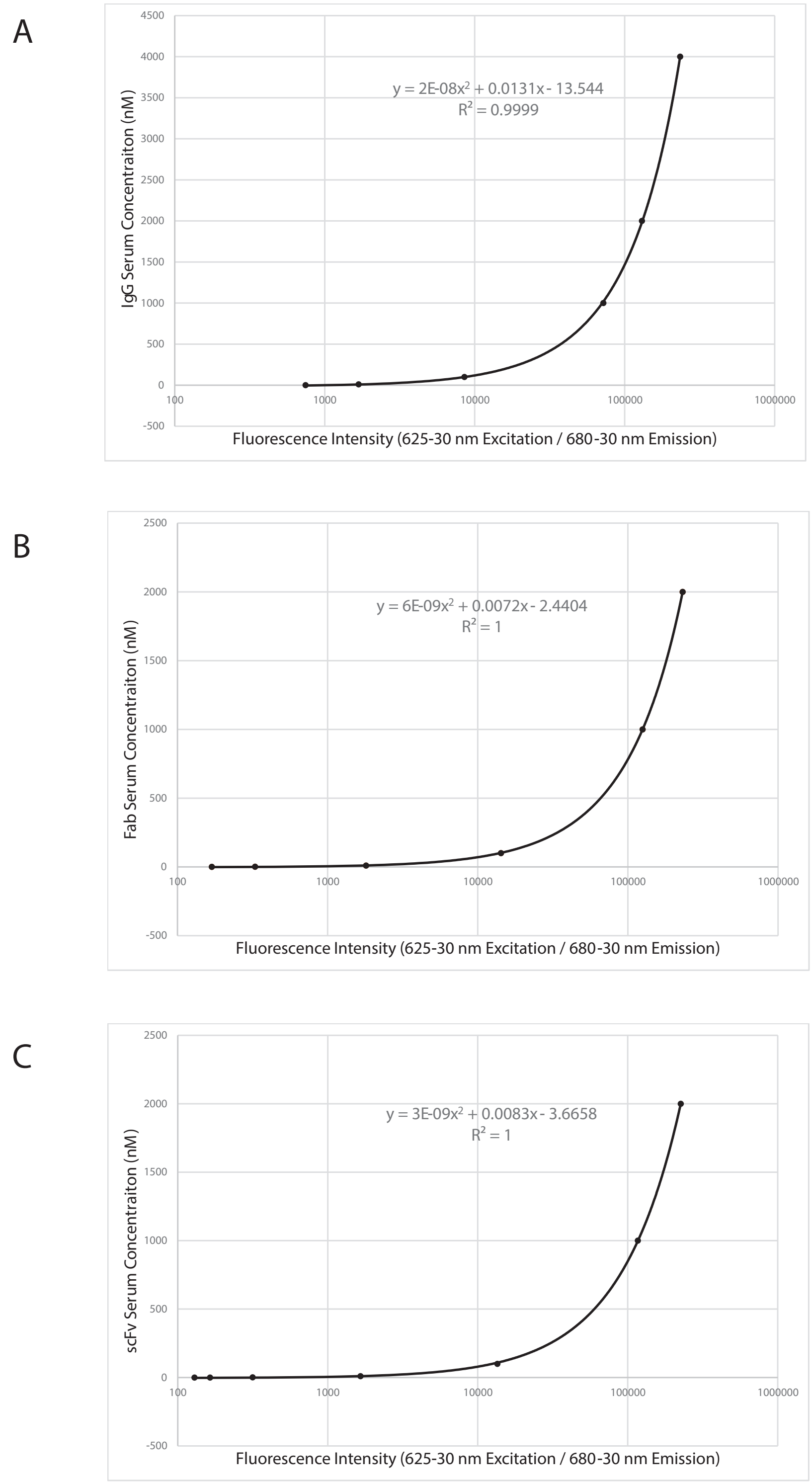

Supplementary Figure 3: Standard curves of fluorescence intensity used to calculate RN2N (A) IgG, (B) Fab and (C) scFv serum concentration.
